# Demand for *Taenia solium* Cysticercosis Vaccine: Lessons and Insights From the Pig Production and Trading Nodes of the Uganda Pig Value Chain

**DOI:** 10.3389/fvets.2021.611166

**Published:** 2021-04-27

**Authors:** Emily Ouma, Michel Dione, Nadhem Mtimet, Peter Lule, Angie Colston, Samuel Adediran, Delia Grace

**Affiliations:** ^1^Policies, Institutions and Livelihood Program, International Livestock Research Institute, Kampala, Uganda; ^2^Animal and Human Health Program, International Livestock Research Institute, Dakar, Senegal; ^3^International Fund for Agricultural Development (IFAD), Cairo, Egypt; ^4^Global Alliance for Livestock Veterinary Medicines, Nairobi, Kenya; ^5^Impact at Scale Program, International Livestock Research Institute, Addis Ababa, Ethiopia; ^6^Animal and Human Health Program, International Livestock Research Institute, Nairobi, Kenya; ^7^Food Safety Systems, Natural Resources Institute, University of Greenwich, Kent, United Kingdom

**Keywords:** *T. solium* cysticercosis, TSOL18 vaccine-oxfendazole, choice experiments, pigs, Uganda

## Abstract

*Taenia solium* cysticercosis disease remains a key challenge to the pig sector in low- and middle-income countries in sub-Saharan Africa, Latin America and South East Asia, resulting in both economic losses and public health impacts. The World Health Organization has ranked it first on the global scale of foodborne parasites. A One Health approach has been recommended for reduction of infection pressure and eradication in the longer term. A new vaccine TSOL18 (Cysvax™), applied in combination with oxfendazole (Paranthic 10%™), a dewormer drug has been developed and field tested for the control of *T. solium* cysticercosis, with high potential to break the disease cycle. It is however unclear whether the products can be marketed through a market driven approach, and if smallholder pig farmers would be willing to take up and pay for the vaccine–oxfendazole combination. A choice experiment methodology was used to assess the potential demand and willingness to pay for the vaccine—oxfendazole combination by Ugandan smallholder pig farmers, and demand for vaccinated pigs by pig traders. The results showed that farmers highly valued quality assurance attributes and were not keen on the vaccine if there were no associated returns in the form of premium price for vaccinated pigs during sales. They were willing to pay US$ 2.31 for the vaccine if it resulted in a premium price for vaccinated pigs. Furthermore, they preferred an accompanying vaccine viability detector as part of its quality assurance. The pig traders on the other hand preferred high carcass weight of pigs, potentially achieved by using oxfendazole. The results show that unless the pig market systems pay a premium price for vaccinated pigs, and quality assurance systems guarantee quality vaccine, uptake of the TSOL18 vaccine and oxfendazole by farmers through market mechanisms may be unsuccessful. The current pig marketing system does not reward food safety, the focus is mainly on carcass weight. Alternative delivery mechanisms for the vaccine through a mix of private–public investments needs to be explored, as the benefits of vaccinated pigs are societal and include reduction and elimination of neurocysticercosis in the long run.

## Introduction

Zoonotic parasites, such as *Taenia solium* cysticercosis are a key challenge to the pig sector in low- and middle-income countries in sub-Saharan Africa, Latin America and South and South East Asia, resulting in both economic losses and public health impacts. Despite its traumatizing health and socioeconomic impacts, *T. solium* cysticercosis has received little attention in terms of investments for control and elimination and is considered a neglected disease by The World Health Organization (1), despite being a potentially eradicable disease. WHO has ranked *T. solium* cysticercosis first on the global scale of foodborne parasites. Eradication of *T. solium* cysticercosis is deemed feasible, because there exists efficient intervention strategies which can interrupt the parasite life cycle (2). Yet, cysticercosis is still endemic in most countries of Latin America, Asia, and Africa. Hotez et al. (3) attributes this to the fact that it is a disease affecting the poor, referring to it as a “forgotten disease of forgotten people” which does not motivate governments to take the necessary measures. A One Health approach involving several efforts at the household, herd, community and national levels, by medical, veterinary, environmental, policy and social sectors has been recommended as the best intervention toward the control and eventual elimination of *T. solium* cysticercosis and taeniasis (4). However, the feasibility of delivery of interventions in whole or part through private or public sector investments remain unclear. This will largely depend on the context and circumstances of various countries.

Humans are the definitive hosts of *T. solium* and harbor the adult tapeworm. Infections result from ingestion of raw or undercooked pork infected with active *T. solium* larval cysts, resulting in taeniasis in humans (5). Pigs act as intermediate hosts, acquiring *T. solium* cysticerci, (the larval stage of tapeworm) in their tissue, through the ingestion of *T. solium* eggs shed in the feces of humans suffering from taeniasis (6). The pigs get infected by consuming the human feces or water or feed contaminated with tapeworm eggs from humans. Humans can also harbor the cystic stage in their tissue following ingestion of *T. solium* eggs through food, water, or surfaces contaminated with feces (7). The *T. solium* eggs develop into cysts in different body tissues with serious consequences resulting from cysts lodged in the central nervous system, a condition termed as neurocysticercosis. Neurocysticercosis leads to various neurological symptoms, most commonly epileptic seizures and chronic headaches.

*T. solium* is suspected to be present in all sub-Saharan Africa countries with a prevalence of 0–14% for human *T. solium* taeniasis and 0.68–34.5% for *T. solium* cysticercosis depending on the region, study population, and diagnostic technique used (8). Studies such as Assana et al. (9) and Gabriël et al. (10), among others, have shown that poor living conditions coupled with poor management of pig husbandry in rural communities in developing countries greatly contribute to maintain the life cycle of the parasite between humans and pigs. Yet, at least 80% of people with epilepsy in the world live in resource-poor countries where most of them are affected by neurocysticercosis (11). The risk factors associated with *T. solium* cysticercosis include low standards of personal hygiene, poor environmental sanitation with inadequate disposal of containment of human stool, poor pig management particularly widespread occurrence of free roaming pigs, lack of and/or inadequate meat inspection, absence of control measures at all levels of the market chain and general lack of knowledge (12).

Due to paucity of good quality data, very few studies have estimated the economic impact of *T. solium* cysticercosis both from the public health and agriculture sector perspectives. Economic losses in the public health sector are associated with human cysticercosis, particularly neurocysticercosis. Gabriël et al. (4) and Hay et al. (13) show that neurocysticercosis is responsible for 30% of acquired epilepsy in endemic areas. Praet et al. (14) reported an estimated total annual cost due to *T. solium* cysticercosis of over 10 million euros resulting from direct and indirect losses, mainly from neurocysticercosis in west Cameroon. Other studies such as Murray and Lopez (15) in their estimation of the Global Burden of Diseases show that 503,000 Disability Adjusted Life Years (DALYs) were related to cysticercosis in 2010. The losses in the agricultural sector are largely due to reduced value of infected pork and carcass condemnation. Zoli et al. (16) estimate annual losses due to *T. solium* cysticercosis in 10 western and central African countries to be more than 25,000,000 Euros. Annual losses due to *T. solium* cysticercosis in Cameroon alone have been estimated to be a minimum of 2,000,000 Euros based on a loss of 30% of the value of the carcass (17). In most of the low- and middle-income countries, there is lack of well-organized meat inspection and official slaughter facilities, thereby partial or total condemnation of carcasses due to cysticercosis is rather exceptional and a high percentage of infected carcasses are marketed and consumed.

Over the past decade research has been undertaken to develop practical vaccines for use in pigs to prevent transmission of *T. solium*. A new vaccine TSOL18 (Cysvax™), applied in combination with oxfendazole (Paranthic 10%™), a dewormer drug has been developed and tested for the control of *T. solium* cysticercosis, with high potential to break the disease cycle. More recently, TSOL18 has been proven to be highly effective against naturally acquired infection with *T. solium* in pigs. Application of TSOL18 has been shown to be highly effective at complete elimination of *T. solium* pig infections during field trials when both primary and booster vaccines are applied in combination with oxfendazole treatment (18). Primary vaccination is given to pigs at least 2 months old, and the booster may be given 3 months after the primary vaccine. Oxfendazole eliminates the cysts that are already lodged in the pigs before vaccination and is also effective against other internal parasites and worms in the pig. Immunity in pigs develop within 2 weeks of the booster dose. In 2013, oxfendazole manufactured under Good Manufacturing Practice (GMP) standards was licensed for the first time for use in pigs to treat cysticercosis, while the TSOL18 vaccine was licensed in 2016 in India. Field trials to assess the efficacy of the combined use of the vaccine and oxfendazole in enhancing immunity against *T. solium* have been implemented in several countries including Uganda (19), Cameroon (20), and Nepal (21). Results from the trials have confirmed the efficacy of the vaccine and oxfendazole package. It is however unclear whether the products can be marketed through a purely market driven approach, and if pig farmers would be willing to take up and pay for the vaccine and oxfendazole package. Several studies, for example Karanja-Lumumba et al. (22), have shown that the propensity of poor, smallholder farmers to invest in preventative animal health treatments, even highly effective ones, may be very low, potentially undermining a purely market driven approach.

In Uganda, the pig sector has grown in the last decade. Demand for pork is increasing rapidly and the annual per capita pork consumption, at 3.4 kg, is the highest in East Africa (23). Fueled by the increasing demand, the number of pigs increased from 0.2 to 4.1 million between 1980 and 2018 (24). Most of the pigs are raised under smallholder systems characterized by poor husbandry practices. The prevalence of *T. solium* cysticercosis is high in several parts of the country. A field survey conducted in 2000 reported an average prevalence of 24% in five districts in the Lake Kyoga basin (25). However, in high pig density areas such as Masaka, Mukono and Kamuli districts, that are also characterized with better sanitation, prevalence is lower, estimated at 11–13% (26). We utilize a choice experiment methodology to assess the potential demand for the vaccine by the Ugandan smallholder pig farmers and their preferences for the technical and administrative attributes of the vaccine and oxfendazole package. We also assess demand for *T. solium* cysticercosis vaccinated pigs by pig buyers and examine the implications of the results on the delivery mechanism of the products through either public or private sector efforts. Both the TSOL18 vaccine and oxfendazole are not yet available for production in Uganda.

## Materials and Methods

### Choice Experiments

The choice experiment framework used in this study is based on a multi-attribute stated preference method that assesses the value of single attributes of a bundled good such as a vaccine, by using individuals stated preference in a hypothetical scenario (27). Preferences are measured directly, and then related to utility, making it possible to estimate economic values of attributes of the vaccine and willingness to pay for vaccine options. Its theoretical framework derives from the Lancasterian consumer theory and discrete choice random utility theory (28). The vaccine attributes, and attribute levels are identified and combined according to an experimental design to create sets of discrete choice alternatives. Respondents are then presented with a series of choice alternatives and asked to choose their most preferred option. Each choice alternative is characterized by several attributes, one of which is a monetary attribute offered at different levels across alternatives. Analysts can then assess how respondents' choices change as the attributes and monetary amounts are varied. Appropriate models are then applied to the choice data to reveal a measure of utility for the attributes of the choices.

Choice experiments have been used in a few studies to assess decision-making by livestock keepers regarding vaccination of livestock to help inform vaccine development and policy. Bennett and Balcombe (29) implemented choice experiments to assess cattle farmers' attitudes to and willingness to pay (WTP) for a bovine tuberculosis cattle vaccine. Terfa et al. (30) employed a discrete choice experiment approach to elicit farmers' preference for attributes of Newcastle disease vaccination programs for village poultry systems. Other studies such as Railey et al. (31) examined household preferences for accurate and timely vaccine information delivered through diagnostic testing to inform which Foot and Mouth Disease vaccine to apply during an outbreak.

In this study, we applied the choice experiment at two levels. The first level focused on farmers preferred vaccine attributes and willingness to pay for vaccine options. The second level focused on pig traders' attributes for slaughter pigs and willingness to pay for vaccinated pigs. The traders purchase pigs for slaughter from farmers. The vaccine and pig attributes and the associated attribute levels used in this study were identified based on previous studies and expert opinion. Six key vaccine attributes covering both technical, administrative features and effect were identified. The technical attributes were inclusion of a vaccine viability detector and frequency of vaccination to attain pig immunity. The vaccine viability detector is a monitor included on vials containing the vaccine and gives a visual indication of vaccine potency. The vaccine viability indicator attribute was identified as important in providing confidence to the users of the vaccine. The frequency of vaccination to attain pig immunity was identified as important as it depends on the period that pigs are reared on-farm, which depends on the type of production system practiced. Poudel et al. (21) indicate that primary vaccination is given to pigs at a minimum age of 2 months old and a booster vaccine given 3 months after the primary vaccine. Immunity develops within 2 weeks of the booster dose. Weaner pigs in farrow-wean systems may spend <5 months on-farm.

The administrative features of the vaccine were identified as the price or cost of the vaccine to the farmer and the vaccine administration cost. The vaccine effects were price premium for the vaccinated pigs and pig liveweight gain due to the oxfendazole de-wormer effects. The cost of the vaccine per dose was computed based on the manufacturer's cost of producing the vaccine, freight, insurance and delivery charges to the warehouse, transport costs to retail outlet, and a markup price by the retailing veterinary stockists. The administration cost of the vaccine included a service fee for the veterinarian/animal health worker without including transport. The vaccinated pig attributes for the traders' choice experiment included carcass weight gain, proof of pig vaccination, market price of pig and the premium price due to vaccination. For each attribute, two or three levels were identified as presented in Table 1.

**Table 1:** TSOL18 vaccine and oxfendazole attributes.

**Attribute**	**Levels**
A. Cost of vaccine which includes the cost of two doses of oxfendazole and TSOL18 vaccine	0. UGX10,500 (US$2.9) 1. UGX13,500 (US$3.8) 2. UGX18,000 (US$5.0)
B. Administration of vaccine which includes service fee for the veterinarian/animal health worker without including transport)	0. UGX2,500 (US$0.7) per pig—service fee for veterinarian or animal health worker who administers vaccine and deworming service to a group of 10 farmers
	1. UGX4,000 (US$1.1) per pig—service fee for an animal health worker who administers vaccine and deworming service to one farmer
	2. UGX6,000 (US$1.7) per pig—service fee for veterinarian who administers vaccine and dewormer to one farmer
C.Improved pig weight gain	0. Pig gains an extra 10% weight because other worms are killed by the dewormer
	1. Pig gains an extra 5% weight because other worms are killed by the dewormer
D. Top up price premium for vaccinated pigs	0. 50% of market price 1. 30% of market price 2. 15% of market price
E. Frequency of vaccination to attain immunity	0. Once at 2 months old
	1. Twice (one dose at 2 months old and another dose 3 months after)
	2. Three times (one dose at 2 months of age, second dose 3 months later, and a third dose after another 3 months)
F. Vaccine viability detector	0. Non-inclusion of an indicator to test for vaccine viability
	1. Inclusion of indicator that shows vaccine viability

*Exchange rate: US$1 is equivalent to UGX 3600 during the study period*.

Attributes associated with vaccinated pigs were also identified using the same process. Four key attributes were identified as presented in Table 2.

**Table 2:** Attributes for vaccinated pigs.

**Attribute/Trait**	**Level**
A. Top-up premium price due to *T. solium* cysticercosis-vaccinated pig	0. 5% top-up 1. 10% top-up 2. 15% top-up 3. 20% top-up
B. Market price of pig (average of a 40-kilogram liveweight pig)	0. UGX155,000 (US$43.1)
	1. UGX200,000 (US$55.6)
	2. UGX225,000 (US$62.5)
	3. UGX250,000 (US$69.4)
C. Proof of vaccination	0. Producer's word
	1. Certificate provided by a government veterinarian
	2. Certificate provided by a private veterinarian
	3. Vaccinated pigs are ear-tagged
D. Improved carcass weight gain	0. Pig gains an extra 15% carcass weight because other worms are killed by the dewormer
	1. Pig gains an extra 10% carcass weight because other worms are killed by the dewormer
	2. Pig gains an extra 5% carcass weight because other worms are killed by the dewormer

*Exchange rate: US$1 is equivalent to UGX 3600 during the study period*.

**Figure 1:**
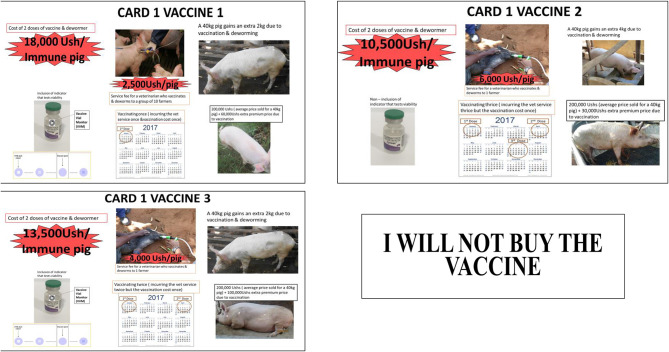
An example of a choice set option for farmers.

The identified attributes and the associated levels (farmer level survey) were combined based on a fractional factorial orthogonal main effects-only experimental design using SAS software (32). The design resulted in 12 generic vaccine choice sets, each with three alternatives and a “no-buy” option. The choice sets were used to construct choice cards with pictorial profiles describing the differences in vaccine attributes and levels to demonstrate each choice set to the farmer respondents. The 12 vaccine choice sets were blocked into two groups of six choice sets each. Each respondent was presented with six choice sets. Figure 1 shows an example of a choice set option presented to the farmers.

**Figure 2:**
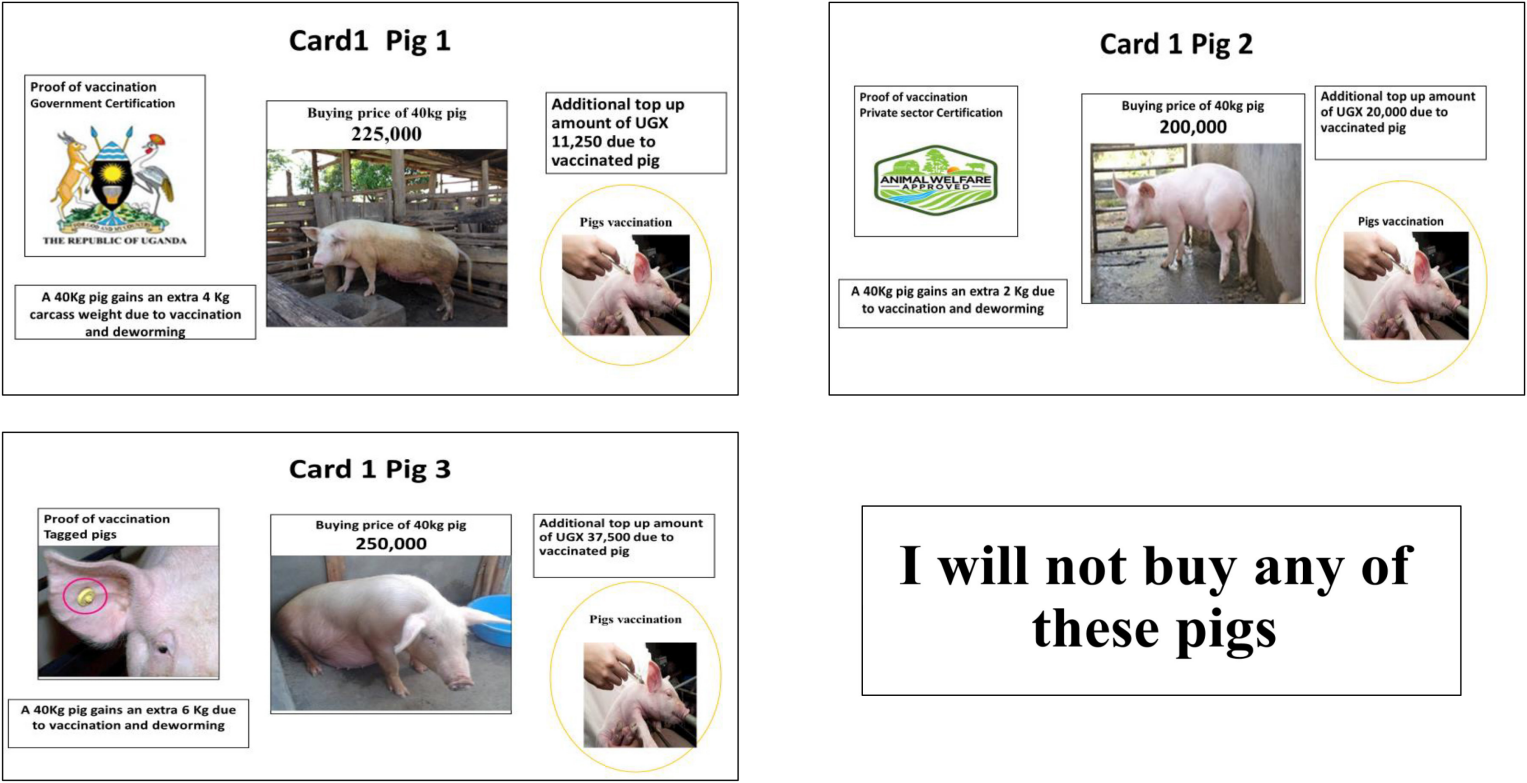
An example of a choice set option for traders.

The experimental design for vaccinated pig attributes (pig trader survey), also using a fractional factorial orthogonal main effects-only experimental design (SAS software), resulted in 8 choice sets, each with three alternatives and a “no-buy” option. The eight choice sets of vaccinated pigs were all presented to the pig trader respondents. Figure 2 shows an example of a choice set option presented to the traders.

The overall efficiencies of the experimental designs were high; D efficiency: 98.6%; A efficiency: 97.1%; and G efficiency: 93.4%. The high efficiencies show that the designs are statistically efficient. The key consideration is that maximizing statistical efficiency minimizes the variability of the parameter estimates (33).

### Implementation of the Choice Experiments

The choice experiment was administered as part of a short farm-level and pig traders survey questionnaire using in-person interviews. The surveys were conducted between November and December in 2018. The farmers survey tool included the choice cards with pictorial profiles describing the vaccine and pig attributes. The rest of the questionnaire covered socioeconomic aspects such as location of the farm and other household- and farm-level characteristics. The pig traders survey tool was similar to the farmers survey with the exception of some specific questions about the traders' business activities and their perceptions and attitudes regarding the role of various actors in the control of porcine cysticercosis.

The administration of the choice experiment was conducted in the following manner, the respondents were first asked if they were aware of *T. solium* cysticercosis, and its effects. They were then provided with a background on *T. solium* cysticercosis and its transmission cycle and health effects. They were also provided with information about the *T. solium* cysticercosis vaccine that may soon be introduced in the market in Uganda and the importance of feedback from pig farmers and pig traders to the vaccine manufacturer. They were then presented with the choice cards developed from the information in Tables 1, 2 in the form of pictorial profiles. The farmers were shown three vaccine choice options at a time for each of the six choice sets and asked to choose the most preferred vaccine option to purchase. Similarly, the traders were shown three choice options of vaccinated pigs for each of the eight choice sets. In each case, a “no-buy” option was also presented for farmers and traders who preferred none of the three options.

### Choice Experiment Modeling

A utility maximizing behavior is assumed, implying that the probability of a decisionmaker, *n* choosing vaccine choice alternative *A*, from a finite set of *j* alternatives in a choice set *k*, occurs if and only if it yields higher utility compared to any other alternative. This is depicted as;

(1) $$\begin{array}{r} P\left(A\right)=Prob\left(V_{nA}+\varepsilon_{nA}>V_{nj}+\varepsilon_{nj}\right)A\neq j,\;\forall j\in k \end{array}$$

P(A): probability of choosing alternative A

*V*_*nj*_: deterministic component of the utility

ε_*nj*_: stochastic component of the utility

Rearranging Equation 1 yields;

(2) $$\begin{array}{r} P\left(A\right)=Prob\left(\varepsilon_{nj}-\varepsilon_{nA}<V_{nA}-V_{nj}\right) \end{array}$$

The distributional assumptions on ε leads to various choice models.

We used a mixed logit model using NLOGIT 6 econometric software to assess factors that influence choice and to estimate the willingness to pay for the vaccine attributes, the vaccine options and vaccinated pigs. From Equation 1, the utility associated with vaccine choice alternative *A* as evaluated by each individual decisionmaker *n* is represented in a discrete choice model by a utility expression *U*_*nA*_ of the general form;

(3) $$\begin{array}{r} U_{nA}=\beta_{n}V_{nA}+\varepsilon_{\;nA} \end{array}$$

Where V_nA_ is a vector of observed variables that includes the attributes of the vaccine and vaccinated pigs, and socioeconomic characteristics of the respondent, β_n_ is the taste coefficient vector associated with V_*nA*_, for respondent *n* and ε_nA_ is an unobserved stochastic term that is assumed to be identically and independently distributed with a Gumbel distribution. The coefficients β, vary over respondents in the population with density *f(*β*)*. The density is a function of parameters Θ that represent the mean and covariance of the β's in the population (28). The vector of random coefficients β can be expressed as the population mean and the individual specific parameter deviation from that mean. The decision makers know the value of their own β_n_ and ε_nA_ for all *j* alternatives and chooses alternative *A* if and only if it is greater than the other choice alternatives. Conditional on β, the probability that the decisionmaker selects alternative A results in the choice probability;

(4) $$\begin{array}{r} P_{nA}\left(\beta_{n}\right)=\;\frac{e^{\beta_{n}V_{nA}}}{\sum_{j}\beta_{n}V_{nj}} \end{array}$$

However, β_n_ is unknown to the analyst we therefore used the unconditional probability. The unconditional probability is the integral of the conditional probability in equation (4) over all possible values of β which depends on the distribution of β, that is unknown to the analyst. This takes the form of a mixed logit probability:

(5) $$\begin{array}{r} P_{nA}=\int \left(\frac{e^{\beta_{n}V_{nA}}}{\sum_{j}\beta_{n}V_{nj}}\right)f\left(\beta \right)d\beta \end{array}$$

We assumed a normal distribution for the taste coefficients, β. Since the integrals in Equation 5 do not have a closed form, it is simulated by taking draws of β from the population density *f(*β*)*|Θ. In this study, Halton draws, which yield much more accurate approximations in Monte Carlo integration relative to standard pseudo-random draws, are used (28). The implicit prices or willingness to pay (WTP) for the vaccine and vaccinated pigs attributes is estimated as the rate of change in the attribute divided by the rate of change of the cost attribute, also referred to as the marginal rate of substitution. This is represented as:

(6) $$\begin{array}{r} WTP_{n}=\frac{\partial U/{\partial Z}_{nj}}{\partial U/{\partial P}_{nj}}=-\frac{\beta_{n}}{\gamma_{c}+\gamma_{a}} \end{array}$$

*P* is the cost associated with the vaccine and includes both the cost of buying the vaccine and its administration cost, as represented by the coefficients γ_*c*_ and γ_*a*_, respectively. The confidence intervals of these non-linear functions of parameter estimates, was approximated using delta method.

The choice experiment variables used in the model and the coding of their corresponding levels are presented in Table 3. We employed dummy variable coding for the choice experiment variables to measure non-linear effects in the attribute levels. The dependent variable in the mixed logit model is a dummy variable showing the choice option selected by each respondent for any given vaccine or vaccinated pig choice alternative.

**Table 3:** Choice experiment variable coding.

**Independent variables**	**Units and coding of the variable levels**
**Vaccine attributes mode**	
Cost of vaccine	Cost in US$
Premium price	% top up of market price
Low vaccination frequency	1 = Once at 2 months, 0 otherwise
Medium vaccination frequency	1 = Twice in the life of the pig, 0 otherwise
High vaccination frequency^a^	1 = Thrice in the life of the pig, 0 otherwise
Weight gain	% of weight gain in the pig due to the dewormer
Vaccine viability detector	1 = Inclusion of a vaccine viability detector, 0 otherwise
Vaccine administration cost	Cost in US$
**Vaccinated pig attributes model**	
Market price of 40 kg liveweight pig	Price in US$
Top-up premium price	% increase due to pig vaccination
Proof of vaccination—private vet certificate	1 = Yes, 0 otherwise
Proof of vaccination—government certificate	1 = Yes, 0 otherwise
Proof of vaccination—ear tagging^a^	1 = Yes, 0 otherwise
Proof of vaccination—producer's word	1 = Yes, 0 otherwise
Improved carcass weight	% increase in carcass weight due to deworming

a *used as the base scenario in the model*.

### Study Area and Sample Size

**Figure 3:**
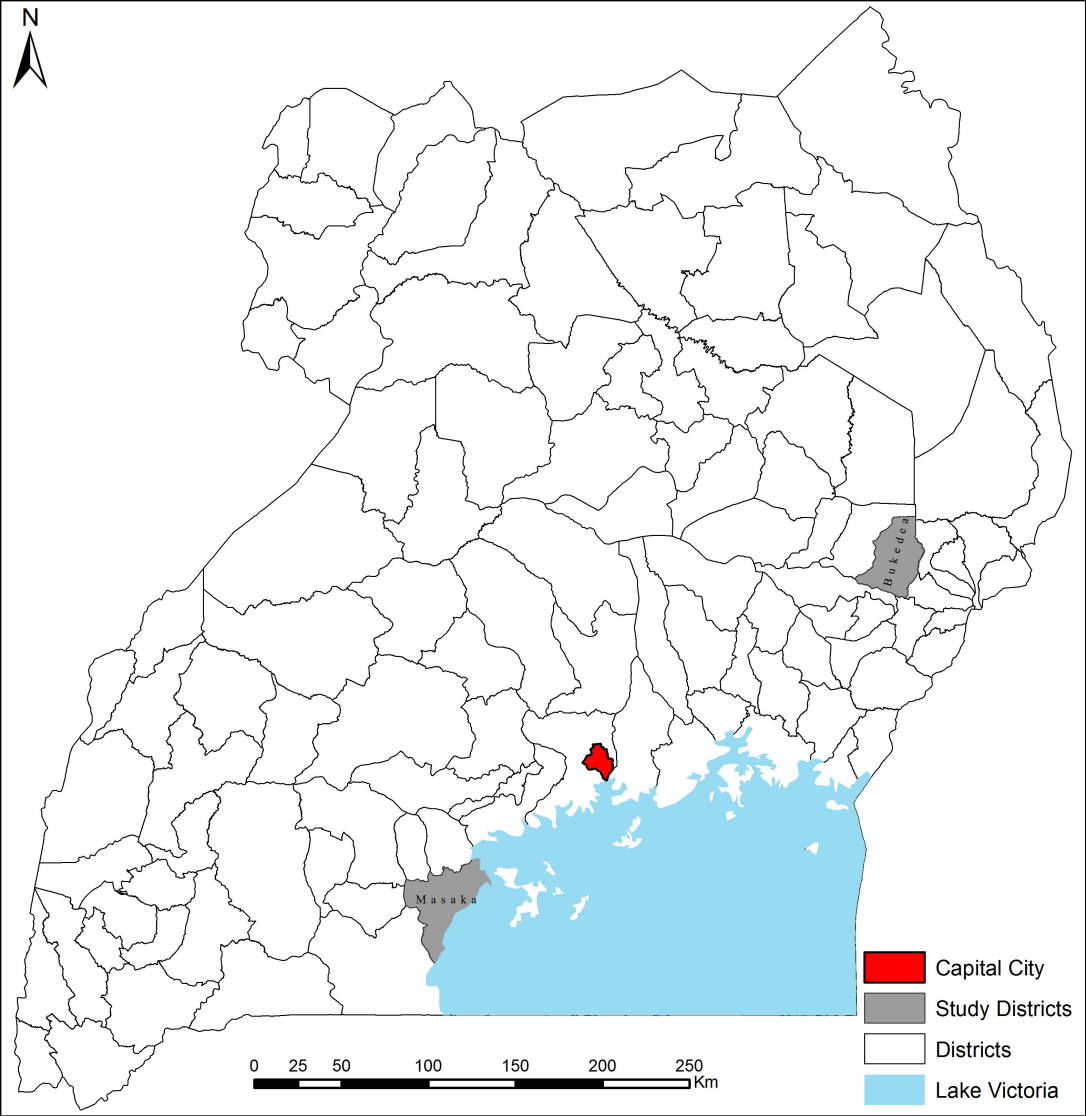
Location of the study sites.

The study took place in two districts of Uganda, Masaka and Bukedea (Figure 3). Masaka is in central region and was selected because it has the highest pig population density in the country. Several pig value chain projects also operate in the district. Bukedea is in the eastern region and was selected because *T. solium* cysticercosis vaccine trials were carried out by the Global Alliance for Livestock Veterinary Medicines (GALVmed) in the district^1^. Some of the pig value chain actors in Bukedea district were therefore aware of the vaccine. The selection of the two districts was therefore to leverage on existing information on vaccine trials and *T. solium* cysticercosis awareness.

A total of 294 pig farmers from Masaka and Bukedea districts participated in the *T. solium* cysticercosis vaccine choice experiment interviews. Forty eight percent of the farmers were from Masaka and 52% from Bukedea. Each farmer responded to six choice sets, yielding a total of 1,764 observed choices. A total of 33 pig traders from Bukedea district participated in the vaccinated pig choice experiment interviews. Each trader responded to eight choice sets, yielding a total of 264 observed choices.

## Results

### Awareness of *T. solium* Cysticercosis and Perceptions on Practices and Roles of Various Actors for Control

At least half of the pig farmers interviewed were aware of *T. solium* cysticercosis. Eighty percent of those who were aware of it were from Bukedea district. Their main source of information was the area veterinary officer or animal health assistant. Twenty-two per cent of the farmers in the overall sample indicated their pigs had suffered from *T. solium* cysticercosis in the last 24 months. This resulted in loss of pig income as most of the traders offered lower price for the pigs once they discovered the animals had cysts.

Eighty per cent of the traders surveyed indicated they had come across *T. solium* cysticercosis infected pigs. They recognize the disease mainly by lingual palpation, checking below the tongue of the pigs for cysts. Most of the traders, 94% indicated rejection of pigs suffering from *T. solium* cysticercosis, though during periods of pig scarcity they sometimes purchase infected pigs at lower prices. Table 4 shows traders' perceptions about practices and roles of various actors on control of *T. solium*. The traders reported being concerned about consumers health and perceive the vaccine as the most effective control option. They however believe that controlling *T. solium* cysticercosis is the role of government and should therefore subsidize the cost of the vaccine.

**Table 4:** Pig traders' perceptions on practices and roles of various actors for control.

**Statement**	**Level of agreement (% of respondents)**				
	**Strongly disagree**	**Disagree**	**Neutral**	**Agree**	**Strongly agree**
I believe it is important to protect my consumers' health by ensuring that I sell *T. solium* cysticercosis-free pigs/pork	9.1	0.0	0.0	30.0	60.6
I condemn pork/pigs infected with *T. solium* cysticercosis	3.0	0.0	0.0	24.2	72.7
The market system should encourage farmers to vaccinate their pigs against *T. solium* cysticercosis by giving premium prices	3.0	0.0	6.1	69.7	21.2
I believe the *T. solium* cysticercosis vaccine + dewormer is the most effective option for controlling *T. solium* cysticercosis	0.0	0.0	6.1	51.5	42.4
I feel that control of *T. solium* cysticercosis is the role of the government and it should therefore subsidize the cost of the vaccine	3.0	12.1	3.0	39.4	42.4
Public health is the role of the government, not the pig traders	27.3	3.0	0.0	33.3	36.4
I do not care about *T. solium* cysticercosis-infected pigs because I don't consume them. The consumer is the one to care	66.7	33.3	0.0	0.0	0.0

### Pig Farmers Attribute Preferences for *T. solium* Cysticercosis Vaccine

The mixed logit model results for the *T. solium* cysticercosis vaccine is presented in Table 5. We performed a likelihood ratio test using the conditional logit model estimates as the restricted model and the mixed logit model estimates as unrestricted. The chi-statistic $[\chi_{\left(12,\;0.01\right)}^{2}$ = 26.22] with *p* < 0.001, showed a better model fitness with mixed logit, which allows for random taste variation.

**Table 5:** Mixed logit model estimates for *T. solium* cysticercosis vaccine attributes.

**Parameter**	**Coefficient**	**Standard Error**
**Random parameters in utility functions**		
Vaccine viability detector	0.734***	0.113
Vaccine administration cost	−0.255***	0.068
Cost of vaccine	−1.230**	0.525
**Non-random parameters in utility functions**		
Constant	2.627***	1.056
Squared cost of vaccine	0.000**	0.000
Premium price	1.741***	0.209
Low vaccination frequency	−0.156	0.110
Medium vaccination frequency	−0.114	0.082
Weight gain	−0.139	1.367
**Heterogeneity in mean, parameter variable**		
Vaccine viability: Bukedea	−0.218	0.135
Vaccine administration cost: Bukedea	0.118**	0.058
Vaccine cost: Bukedea	0.256***	0.051
**Standard deviations of parameter distributions**		
Vaccine viability detector	1.549***	0.526
Vaccine administration cost	0.109	0.146
Cost of vaccine	0.243	0.152
Likelihood ratio test^a^	72.35 $\chi_{\left(\chi 12,\;0.01\right)}^{2}$ = 26.22	
Log likelihood function at start values (MNL)	−2445.42	
Simulated log likelihood function at convergence	−2212.38	
Halton draws	100	
Number of observations	1,764	

***, **, and * denotes p-values 1, 5, and 10%, respectively.

The results indicate a strong statistical significance of the mean coefficients of some of the vaccine attributes including vaccine viability detector, administration cost of the vaccine, the cost of the vaccine and premium price of pigs due to vaccination. The model reveals preference for a vaccine that is not costly, has low administration costs, and has a vaccine viability detector integrated. There was also strong preference for the vaccine if farmers get premium price for the vaccinated pigs. Attributes associated with vaccination frequency and weight gain of pigs because of deworming were not statistically significant in the model. The model estimates on cost of vaccine and its administration cost had a significant negative coefficient, confirming the high propensity by farmers to hold onto money as they have high time preference for money.

Associated with each of the mean coefficient estimates of the random taste parameters are derived standard deviations calculated over the 100 Halton draws, indicating the amount of spread that exists around the sample population. The standard deviation of the random coefficient on vaccine viability detector was statistically significant (*p* < 0.01). This implies that different individuals possess individual-specific parameter estimates for that attribute that may be different from the sample population mean parameter estimate. The standard deviations of the other random and non-random parameters were not statistically significant, implying homogeneous parameter estimates for those attributes in the sample population. The constant parameter representing the no-buy options (alternative specific constant terms) was positive and significant (*p* < 0.01) indicating a positive preference for this option. The heterogeneity in mean parameter estimates was statistically significant for the interaction term between vaccine cost and Bukedea district dummy variable at *p* < 0.01. This shows that the differences in marginal utilities for the vaccine cost attribute may be, in part explained by the farmer location effects. This is presented in Table 6 which shows the differences in the random parameter coefficients across the two districts. The coefficients for vaccine viability detector was positive, whereas that for cost of vaccine and vaccine administration cost had a negative sign and were all significantly higher in Masaka compared to Bukedea district.

**Table 6:** Coefficients of mixed logit random parameters, by district.

**Attribute**	**District**		**Mean difference**
	**Bukedea**	**Masaka**	
Vaccine viability detector	0.511 (0.015)^a^	0.727 (0.015)	−0.216***
Cost of vaccine	−0.844 (0.001)	−1.098 (0.002)	0.254***
Vaccine administration cost	−0.118 (0.000)	−0.235 (0.001)	0.117***

a *Standard error in parenthesis*.

*** *Denotes p-values at 1%*.

### Willingness to Pay for *T. solium* Cysticercosis Vaccine

Estimates of the implicit prices of the vaccine attributes are presented in Table 7. The results show two key attributes that were highly valued by farmers: a high premium price for vaccinated pigs and inclusion of a vaccine viability detector. Farmers were willing to pay US$ 1.2 more for the vaccine if it would result in at least 1% market price top up as premium payment for a vaccinated pig. They were also willing to pay US$ 0.5 more if the vaccine comes with a viability detector. This did not differ between Masaka and Bukedea.

**Table 7:** Vaccine attribute implicit prices (willingness to pay values) in United States dollars (US$) and Uganda shillings (UGX).

**Attribute**	**US$**	**UGX**	**Standard error**
Vaccine viability detector	0.495***	1,782	0.189
Price premium	1.173***	4,223	0.429
Low vaccination frequency (once)	−0.105	−378	0.084
Medium vaccination frequency (twice)	−0.077	−277	0.065
Weight gain	−0.094	−3,388	0.923

*** and ** denote significant variables at 1 and 5%, respectively.

We used individual parameter estimates to assess the willingness to pay (WTP) for combined preferred vaccine attributes which includes a vaccine viability detector, a price premium due to vaccination and low vaccination administration costs. This was estimated at US$ 2.31(±0.39) for the overall sample. The WTP estimate was statistically different between Masaka and Bukedea farmers at *p* < 0.01. For Masaka the WTP was US$ 2.37 (±0.41) while in Bukedea it was US$ 2.24 (±0.36).

Table 8 shows the proportion of pig farmers choosing profiles depicting various vaccine options. The base scenario of the attributes is presented in vaccine option 1. This is the scenario that was used to describe the base scenario with an assumption of a price premium from the market for vaccinated pigs. Only 15 % of the surveyed farmers selected that option. The improvement in attributes of the vaccine presented in options 2 and 3 (Table 8) resulted in choice by a higher proportion of farmers. For instance, vaccine option 2 with lower administration cost (US$ 0.69), and a 50% price premium was selected by 37% of the farmers. Vaccine option 3 with 50% price premium, inclusion of a vaccine viability detector and a 10% increase in pig live-weight was selected by 49% of the farmers. The results show that under baseline scenario—only few farmers would be willing to take up the vaccine. Farmers are interested to pay for the vaccine if they are assured of a price premium and have confidence in the quality of the vaccine, through a viability detector.

**Table 8:** *T. solium* vaccine attribute options selected by a high proportion of pig farmers.

**Attributes**	**Vaccine options**		
	**Option 1**	**Option 2**	**Option 3**
Cost of the vaccine (US$)	5.00	2.92	2.92
Vaccine administration cost per pig (US$)	1.67	0.69	1.67
Price premium (% of market price)	15%	50%	50%
Vaccination frequency to attain immunity	Twice	Once	Once
Carcass weight gain (%)	5%	5%	10%
Vaccine viability detector	None	None	Yes
% of farmers choosing the vaccine option	14.9	37.4	48.9

### Traders Preferences for *T. solium* Cysticercosis Vaccinated Pigs

The results of the conditional logit model estimation for *T. solium* vaccinated pigs is presented in Table 9. The results show traders preference for improved carcass weight of pigs (*p* < 0.01). Most of the other variables were not statistically significant, though had the expected coefficient signs.

**Table 9:** Conditional logit estimates for *T. solium* cysticercosis vaccinated pigs.

**Parameter**	**Coefficient**	**Standard error**
Purchase price of pig in USD	0.040**	0.0177
Purchase price—squared	−0.000	0.000
Percent of premium top up price due to vaccination	1.847	1.384
Improved % pig weight gain	4.578**	1.787
Proof of vaccination—farmer's word	−0.194	0.218
Proof of vaccination government veterinarian certification	−0.255	0.373
Proof of vaccination private veterinarian certification	−0.079	0.210
Log likelihood function	−349.469	
Pseudo-R^2^	0.1154	
Number of observations	1,056	

a *Base scenario for proof of vaccination—vaccinated pigs are ear tagged*.

** *Denotes significant variables at 5%*.

## Discussion

Vaccine quality assurance is an important attribute highlighted by the farmers through their high preference for a vaccine with a viability indicator. This depicts a “lemons market” where consumers believe that products in the market are of low quality and will have a low willingness to pay for the product (34). This is usually pronounced when the quality assurance systems are weak, as is the case in Uganda. Pig farmers in Uganda have reported poor performance of products such as drugs and dewormers, which is due to the use of adulterated products, poor handling and misuse (35). Lack of transparency in pig trade, coupled with information asymmetry has been reported at the market level. Therefore, incorporating quality tracers would be of interest to the value chain actors, especially farmers for quality assurance. A similar scenario is reported by a World Bank study on pesticides in Uganda. The World Bank study found that one third of pesticides in the market were sub-standard. However, farmers believed that 40% of the pesticides were sub-standard and this substantially reduced their willingness to pay for pesticides (36). Other studies such as Wane et al. (37), Campbell et al. (38) and Ilukor and Birner (39) confirm the strong linkage between quality of veterinary products and services, and willingness to pay.

The results show preference for attributes associated with low administration cost of the vaccine, as well as cost of the vaccine itself. This confirms the high propensity by farmers to hold onto money as they have high time preference for money. Efforts to reduce transaction costs associated with administration of vaccines through communal vaccination campaigns have been successful in various livestock species. Such efforts can be replicated in this case with careful consideration of control for disease transmission due to mass handling of pigs from different households. The technical feature of the vaccine, requiring more than one vaccination for the pigs to attain immunity contributes to increased expense on the vaccine and the transactions cost associated with its administration. Ideally, one vaccine dose should provide lifetime protection for pigs, since in many production systems the life of a slaughter pig is about 12 months. According to Pedersen et al. (40) this might be possible by using delayed- or pulse-release vaccine formulations or by using live recombinant vaccine vectors.

In this study, pig farmers were willing to pay US$ 2.31(±0.39) for the vaccine with preferred attributes including low administration cost, quality assurance through a vaccine viability detector and premium payment for pigs due to vaccination. This is much higher than what they regularly spend to deworm their pigs—about US$ 1. Paying for a dewormer in combination with the vaccine may be unaffordable for most farmers in Uganda. In countries like Cameroon, pig owners indicated willingness to pay for the TSOL18 vaccine in combination with oxfendazole if the price is affordable (20). Studies such as Geerts (41) report that farmers were not prepared to pay for the vaccine even in areas hyperendemic with *T. solium* taeniasis-cysticercosis. The incentive to invest in the *T. solium* cysticercosis vaccine would be there if quality assurance systems are reliable and the markets provide premium price for vaccinated pigs, an attribute that was highly valued by pig farmers in this study. However, the pig trader results show that markets place more emphasis on carcass weight of the pig. The high carcass weight can be achieved with application of the oxfendazole dewormer to reduce worm burden.

In terms of sustainability of the *T. solium* cysticercosis vaccination efforts, an important consideration raised by Geerts (41) is consideration of who should pay for the vaccine—the pig farmer or the government? The answer to this question depends on whether the vaccine is considered a public good from which the community benefits or a private good from which the farmer benefits. In the case of *T. solium* cystercosis, both benefits. However, the farmers benefit only if they get premium prices, and it is important to note that pig farmers may not be willing to spend more than what they currently do for regular deworming. Results from this study show that the preferred vaccine and oxfendazole product would cost more than double regular deworming, making it unaffordable for farmers. Besides, pig vaccination left to farmers discretion is unlikely to reduce the infection pressure. The World Health Organization (1) considers the “best-bet” option for rapid reduction of infection pressure as a combined approach utilizing the treatment of human taeniasis cases through mass drug administration or selective chemotherapy combined with the vaccination (TSOL18) and treatment of pigs using oxfendazole. This should be supplemented by supporting measures such as health education and measures requiring fundamental social changes including improved meat inspection, improved pig husbandry practices and improved sanitation.

The results indicated that pig traders are aware of the importance of vaccinating pigs and the importance of safeguarding consumers health and safety. However, if there are no incentives mainly through improvements in pig carcass weight, they are not willing to pay a price premium when buying vaccinated pigs. In addition, the traders would not rely on farmers word as proof of vaccination. Considering that smallholders farmers are generally the main pig suppliers to traders (42), there is a need to find alternative ways to increase collaboration and trust between value chain actors and implement a reliable certification system.

## Conclusion

The study analyzed the potential demand for the *T. solium* cysticercosis vaccine package by the Ugandan pig farmers and their preferences for its technical and administrative attributes. From the analysis, unless the pig market systems can pay a premium price for vaccinated pigs, and quality assurance systems guarantee quality of the vaccine, uptake of the package of TSOL18 vaccine and oxfendazole by farmers through market mechanisms may be unsuccessful. Yet, the current pig marketing system does not reward food safety, focus is placed on carcass weight. An alternative option would be for the package of TSOL18 vaccine and oxfendazole to be disseminated through a mix of public and private sector investments as recommended by Thomas et al. (43). The benefits to the community of *T. solium* cysticercosis vaccinated pigs are the decline and eventual disappearance of *T. solium* tapeworm carriers and, in the long term, neurocysticercosis (41). This is sufficient justification for a government to invest in consumer awareness and vaccination campaign against *T. solium* cysticercosis. The findings have implications for livestock diseases of public health significance.

## Data Availability Statement

The raw data supporting the conclusions of this article will be made available by the authors, without undue reservation.

## Ethics Statement

The studies involving human participants were reviewed and approved by Research and Ethics Committee of the College of Veterinary Medicine, Animal Resources and Biosecurity–approval reference VAB/REC/15/127. The participants provided their written informed consent to participate in this study.

## Author Contributions

AC and SA conceived the study. DG, MD, NM, and EO developed the methodologies and tools. PL collected the field data under the supervision of MD and EO. NM and EO conducted the analysis. All authors listed have made a substantial contribution to the work and have approved this submission for publication.

## Conflict of Interest

The authors declare that the research was conducted in the absence of any commercial or financial relationships that could be construed as a potential conflict of interest.

## Footnotes

^1^https://www.galvmed.org/new-tools-tackle-porcine-cysticercosis-rural-uganda/.
